# Using technology for patient-centered care at home after CAR T-cell therapy or stem cell transplant: a prospective feasibility study

**DOI:** 10.3389/fimmu.2025.1403249

**Published:** 2025-06-18

**Authors:** Susan L. Moore, Glen J. Peterson, Sarah R. Montoya, Bellinda K. Conte, Rachel K. Brahler, Carolyn Hutchison, Katherine L. Hoople, Clayton A. Smith

**Affiliations:** ^1^ Division of General Internal Medicine, University of Colorado School of Medicine, University of Colorado Anschutz Medical Campus, Aurora, CO, United States; ^2^ mHealth Impact Lab, Department of Community and Behavioral Health, Colorado School of Public Health, University of Colorado Anschutz Medical Campus, Aurora, CO, United States; ^3^ Division of Hematology, University of Colorado School of Medicine, University of Colorado Anschutz Medical Campus, Aurora, CO, United States; ^4^ Office for the Vice Chancellor of Research, Clinical Research Support Team, University of Colorado Anschutz Medical Campus, Aurora, CO, United States; ^5^ Reimagine Care, Inc., Nashville, TN, United States; ^6^ CU Innovations, University of Colorado Anschutz Medical Campus, Aurora, CO, United States; ^7^ OncoVerity, Inc., Aurora, CO, United States

**Keywords:** remote patient monitoring, digital health, CAR T-cell therapy, bone marrow transplant, stem cell transplant, patient-centered care

## Abstract

**Introduction:**

Febrile neutropenia, neurotoxicity, and cytokine release syndrome are dangerous and damaging side effects seen in more than half of patients with cancer who receive critical chemotherapies or immunotherapies respectively. Early intervention and care can reduce complications, but timely treatment in the outpatient setting is often delayed due to dependency on interval-based, patient-driven self-assessments. Using digital health technologies (DHT) to monitor patients remotely can improve time-to-intervention and health outcomes. Providing follow-up treatment and essential support to patients at home can further reduce patients’ and caregivers’ burden and improve patient satisfaction.

**Methods:**

This pilot feasibility study examined the results of a patient-centered program for technology-assisted remote patient monitoring and symptom reporting for patients undergoing autologous or allogeneic stem cell transplant (SCT) or CAR T-cell therapy. Technical and operational feasibility and user experience were assessed for patients, caregivers, and providers. Ten patients between 30 and 80 years old participated in the study for up to 30 days after CAR T-cell therapy or autologous SCT or up to 90 days after allogeneic SCT. Patients wore biometric sensors around the clock to monitor vital signs and engaged with a chatbot through bidirectional SMS text messages for symptom reporting and regular health check-ins. Virtual care center personnel monitored patient status and followed up with patients or their care providers as needed. Patients, caregivers, and providers completed surveys about their program experience; patients also completed brief interviews.

**Results:**

Nine of 10 patients engaged with DHT based monitoring as intended. A total of 219 alerts were generated, 171 from wearables and 48 from the chatbot and check-ins. Fifty-seven alerts required follow-up with patients, 26 required care team follow-up, and 10 required patients to be seen in a clinical setting. Users found the program acceptable overall, with patients and caregivers reporting perceptions of being more cared for and providers feeling that it improved quality of care. Suggestions received included a desire for more information and improved communication and alerting processes.

**Discussion:**

Overall, DHT-based remote patient monitoring was feasible for use with patients receiving SCT and CAR T-cell therapy. Effective practice integration requires adaptation to clinical workflows. Further evaluation of patient acceptance over time and effectiveness at improving health outcomes is recommended.

## Introduction

Current guideline-based therapy for patients with hematologic malignancies often includes treatments such as stem cell transplant (SCT) or chimeric antigen receptor (CAR) T-cell therapy. While effective, both types of treatment are associated with severe side effects. Standard preparation for SCT requires conditioning chemotherapy, total body irradiation (TBI) in the case of allogeneic SCT, and additional immunosuppressive medications, leaving patients vulnerable to febrile neutropenia (FN) and a variety of infectious complications, including septic shock. As an immunotherapy, CAR T-cell therapy by intent causes immune activation; however, an overly robust immune response can lead to cytokine release syndrome (CRS) and immune effector cell-associated neurotoxicity syndrome (ICANS), which can result in severe illness and life-threatening systemic inflammation, organ dysfunction and multi-organ system failure (1, 2). Over 50% of patients who receive chemotherapies develop FN leading to infection, with a high resultant burden of morbidity in up to 30% and associated mortality of up to 10% (3–5). Among patients who receive CAR T-cell therapy, between 37% and 93% experience some degree of CRS and between 20% and 60% experience ICANS, with severe conditions occurring in up to one-third of patients with CRS and 12 to 30% of those with ICANS (6–10).

Early identification and intervention are critical in managing FN, CRS and ICANS. Standard clinical guidelines for FN recommend administration of antibiotics within one hour of triage, followed by monitoring and supportive care for four hours prior to either discharge back to outpatient care if stable to return home or hospital admission if further treatment is needed (4). Delayed administration increases complications due to FN, which in turn are subsequently associated with increased hospital length of stay, overall morbidity, and elevated risk of 28-day mortality associated with FN with an increase of 18% per hour delay prior to treatment (11). Recommended treatment for CRS is dependent on severity of symptoms and clinical signs. Treatment ranges from continued monitoring and/or antipyretics to IV fluids, oxygen supplementation, vasopressors and immunosuppressive agents such as corticosteroids and tocilizumab. ICANS management is based on severity of symptoms including Immune-Effector-Cell-Associated Encephalopathy (ICE) scoring and additional clinical assessment with treatment including escalating doses of dexamethasone and potentially immunosuppression with anakinra in the most severe cases (1, 2). Careful monitoring and appropriate grading for neurotoxicity management is essential to avoid progression to more advanced stages and unintentional mitigation of the therapeutic response to CAR T-cell treatment (2). However, delays in receiving appropriate treatment are common due to dependence in the outpatient setting on patient-initiated pursuit of care, which includes self-monitoring, accurate and timely identification of symptoms and their severity, follow-up with a healthcare provider, and travel to a clinical setting to receive follow-up care. Additional factors such as diurnal-nocturnal temperature variation, poor patient compliance or technique with self-checks and self-monitoring, and emergence of symptoms while sleeping can also affect timeliness of care (4, 12–16). Care for FN and infectious complications can impede the initiation of conditioning chemotherapy and SCT and interrupt CAR T-cell processes, which can impact the effectiveness of cancer treatment. Furthermore, CRS, ICANS, and FN often contribute to prolonged hospitalizations and/or hospital readmissions leading to significant patient morbidity. Developing an objective, reliable, rapid, and systematic approach to accurately identify elevated temperature, hemodynamic variables and side effect symptoms is key to improve outcomes in the management of FN, infection, neurotoxicity and CRS in SCT and CAR T-cell therapy patients.

Using digital health technology (DHT) to monitor patients and engage patients remotely can improve time to receipt of needed care and improve health outcomes for FN, neurotoxicity, and CRS. Remote patient monitoring (RPM) programs using DHTs such as wearable devices, mobile apps, and text messaging have previously been shown to improve care for patients with cancer and support early detection of symptoms indicative of clinical deterioration (17–25). Bidirectional messaging and interactive electronic patient symptom reporting (ePRO) are observed to increase patient feelings of connection to care and early identification of issues of concern (26). Providing follow-up treatment and essential support to patients through home health management of needs such as intravenous fluids or antibiotics and routine lab tests can further reduce patient and caregiver burden and improve patient satisfaction (27, 28). This study examined the future potential for improving health outcomes among patients who received SCT and CAR T-cell therapy by initially assessing the feasibility of using DHT-assisted support to provide patient-centered oncology care at home.

## Materials and methods

### Study design and care program

This prospective pilot study was designed to assess the technical and operational feasibility of and user experience with an in-home care program (“Cancer Care at Home”) that used RPM and ePRO to support early detection of symptoms and improve treatment for FN, infection, neurotoxicity, and CRS among adult patients who received autologous or allogeneic SCT or CAR T-cell therapy. Prior to initiation, this study was reviewed as human subjects research and approved by the Advarra Institutional Review Board. The study was designed to identify barriers and facilitators among technical and operational processes and user experience considerations that might need to be addressed to successfully scale the intervention in research and practice, and thus was not powered to support conclusions about the program impact on health outcomes at the population level.

The Cancer Care at Home program for the study was provided by Reimagine Care (RC) (29), a third-party technology-enabled health service provider, to study patients who received their oncology care through the UCHealth Blood Disorders and Cell Therapies Center (BDCTC) in Aurora, Colorado. Study patients received program support at home following hospital discharge for up to 30 days (autologous SCT or CAR T-cell therapy) or 90 days (allogeneic SCT) post-treatment. Prior to discharge, patients completed program education with the research team and virtual care team, reviewed their individual care plans, received, activated and affixed the RPM wearable to allow for baseline data collection and confirm patient education on operation. Patients were also introduced during program education to how to use the interactive ePRO system, with messages starting after discharge. RPM devices were to be worn continuously except for the brief periods necessary to recharge the battery (<1 hr charge time approximately every couple of weeks). The ePRO system for the study utilized SMS text messaging interaction with a customized artificially intelligent (AI) chatbot, which was called “Remi.” Messages sent by the chatbot were customized and review of systems (ROS) questions were developed to represent standard medical assessment, with chatbot content development overseen by and approved by the principal investigator (a licensed care provider) in consultation with other members of the care team. Patients were asked to use the chatbot to report their symptoms as needed at any time and respond to health check-in prompts at least weekly.

Advanced practice nurses or registered nurses in RC’s Virtual Care Center (VCC) monitored both RPM and ePRO systems for alerts around the clock. Monitoring was conducted in real-time during normal business hours, and via automated notification of alerts to an on-call system after hours. If an alert was detected, VCC team members first reviewed it to determine if follow-up with patients or with the patient’s clinical team was needed according to standard communication and evaluation protocols established *a priori* with the patients’ clinical team. If an alert required follow-up, VCC team members followed up with patients by telehealth outreach within 15 minutes of the alert to conduct symptom management and independent clinical evaluation, with referral to the patient’s clinical team and additional clinical services including emergency care as needed. If an alert did not require follow-up based on the established protocols, a record of the alert and its disposition was retained in the monitoring system. Patients did not receive alerts directly; notifications to patients were made if needed by VCC team members and/or clinical team members according to established protocols.

Infection-related alerts through wearable RPM used manufacturer-defined criteria, which included 1) elevated skin temperatures greater than 98.5 F or 2.5 times standard deviation from a patient’s own baseline; 2) a mean heart rate greater than 120 beats per minute or 30% over a patient’s own baseline for at least 2 consecutive readings over the course of an hour; and 3) a mean respiratory rate higher than 24 breaths per minute or 30% above a patient’s baseline for at least 2 consecutive readings over the course of an hour in the absence of exertion. A built-in device alert for “infection-like symptoms” was used in addition to alerts for these specific criteria. Patients were also instructed to notify care teams in the event they observed an oral temperature greater than 100.4 F for one hour or a single reading of 101 F or higher while performing twice-daily manual temperature checks as part of their usual care.

Symptom-related alerts through ePRO were based on criteria defined according to 16 discrete management pathways for anorexia, constipation, cough, depression, diarrhea, dyspnea, dysuria, fatigue, fever, hot flashes, insomnia, mouth discomfort, nausea, neuropathy, pain, and rash. Symptoms reported outside these pathways were immediately escalated to an alert. Patients were able to engage with the ePRO chatbot around the clock as they chose, whether in response to prompts or by initiating their own contacts to ask questions or report concerns. Patients received daily prompts for check-ins and a weekly prompt to complete a symptom survey. The ROS assessment asked if patients were experiencing fever or chills, headache or dizziness, chest pain, shortness of breath, a new or worsening cough, new or worsening pain, new or worsening nausea, vomiting or diarrhea, new or worsening swelling of any extremity, and a new or worsening skin rash. VCC team members monitored communications between patients and the chatbot via a dashboard according to a ‘human-in-the-loop’ model that allowed a team member to take over communications from the chatbot at any time.

### Population and setting

Study participants were recruited through the UCHealth Blood Disorders and Cell Therapies Center (BDCTC) in Aurora, Colorado. Patients and their caregivers were eligible for the study if they had received an allogeneic SCT, autologous SCT or CAR T-cell therapy and were stable to be discharged to home for outpatient management according to standard care protocols with the intention of residing within 45 minutes of the BDCTC for the duration of the study; were between 18 and 89 years old; and had in-home caregiver support, reliable home telephone and internet service including a home wireless network, a mobile device (iOS or Android) capable of running study applications, and SMS texting capacity with an unlimited texting plan or other plan able to support study messaging without undue burden. Patients were excluded from study participation if their care providers or the principal investigator believed participation was not in their best interests for clinical reasons or if they were unwilling to wear RPM devices and use the chatbot as indicated.

### Technology systems

The RPM and ePRO services for this study were delivered using commercially available DHTs. RPM used the BioButton Rechargeable^®^ System (BioIntelliSense, Inc.) (30), a FDA-cleared wearable device that supports near real-time collection of physiological data in both home and healthcare settings (31, 32). The wearable is designed for continuous wear (24 hours a day, 7 days a week) with brief periods of removal for recharging needed approximately once every 2 weeks. Data from the wearable are encrypted and securely transmitted through an accompanying mobile phone app. In addition, the BioButton system included an external hub (the BioHub) which connected securely and automatically to the BioButton and acted as a backup data transmission method to the mobile app. A provider-facing dashboard (AlertWatch; provided through BioIntelliSense) with configurable alert thresholds displayed RPM information for monitoring by the VCC team. The ePRO platform (Memora Health) (33) was used to support patient engagement through bidirectional text messages with the AI chatbot for symptom reporting, coordination and management. Text messages from the platform were always delivered to patients from the same number for consistency and trust in the source. Automated guidance was provided using natural language processing (NLP) algorithms, with the VCC team providing human review of patients’ interactions through a provider-facing dashboard. An embedded alerting engine prioritized alerts and escalations to the VCC team members through visual notifications on the dashboard and HIPAA-compliant text messages to VCC team members’ mobile devices as a redundant alerting method.

### Outcomes

Technical feasibility, operational feasibility, acceptability and user experience with the program were assessed as composites through a mixed methods approach using a triangulation design to combine descriptive statistics and qualitative analysis for interpretation. Technical feasibility aspects considered were RPM device wear time, RPM alerts by number and type, number of symptom reports made through the ePRO system (chatbot), and the overall level of technical support required by patients and caregivers (qualitative). Operational feasibility considered patient engagement with the chatbot, number of responses to ePRO prompts, patient use of the ePRO system over the course of the study, number of alerts requiring clinical evaluation and follow-up, and health care utilization. Patient engagement with the chatbot and use of ePRO were assessed qualitatively as well as through descriptive analysis to discern if there was variation in adoption or perception for the three different aspects of the overall ePRO system (chatbot, weekly surveys, ROS check-ins). User experience was assessed qualitatively using data collected through interviews and responses to fixed-choice and open-ended survey items. A threshold of 60% or better on key program outcomes (number of patients who wore RPM devices as intended; number of patients who engaged with the ePRO system as intended) was established to define success.

### Data collection and analysis

Wearable device data was collected around the clock at minimum once per hour. Health care utilization metrics were obtained from patients’ medical records and reports from home health care visits. Symptom reporting data were collected automatically by the ePRO platform. Alert frequency and type depended on established RPM and symptom reporting thresholds. Alert data were extracted from the dashboard databases by Reimagine Care and exported for analysis by the research team. Patients, caregivers, and providers were asked to complete user experience surveys. Surveys were self-administered electronically using REDCap for secure survey data collection (34, 35). Autologous SCT and CAR T-cell therapy patients and caregivers were asked to complete surveys at 14 days and 30 days post-treatment, and allogeneic SCT patients and caregivers were asked to complete surveys at 30 days and 90 days post-treatment. Providers were asked to complete surveys about their experiences with the study program for all patients under their care at the end of the overall study period. Patients also completed brief interviews at the end of the study to explore and describe their experiences in their own words, giving them the opportunity to elaborate on their survey responses. Patients were asked about their overall perceptions of the program, the usability of the technologies, their likes and dislikes, and suggestions for future improvement. Interviews were recorded and transcribed using AI transcription (Otter.ai, Mountain View CA) with human adjudication for error correction and quality control.

Fixed-choice survey items and alert data were analyzed using descriptive statistics. Due to the size of the data set, utilization data were assessed qualitatively. Open-ended survey responses and interview data were evaluated through rapid content analysis according to a method developed by the research team using a dual-read approach conducted by an experienced qualitative analyst, in which the first review heuristically identified emergent topics and the second review identified themes across respondents. Midpoint and endpoint surveys were analyzed together, as most people answered either one or the other but not both. If an individual replied to both midpoint and endpoint surveys, the most recent survey was included in the dataset for analysis. This allowed evaluation using data obtained after the longest duration of experience with the program while avoiding overrepresentation in the dataset.

## Results

A total of 10 patients participated in the study between April and August 2023. The majority of study patients were female (n=6), and all were over 30 years old, with most 50 years or older (n=7). Seven had stem cell transplants (4 autologous, 3 allogeneic) and 3 received CAR T-cell therapy, as summarized in **Table 1**. Six out of 10 patients, 6 of 10 caregivers, and 8 of 11 providers responded to at least 1 survey either at midpoint or at the end of the study period. Eight patients participated in interviews to provide in-depth feedback, the results of which augmented the findings from user experience surveys. One patient declined to complete an interview, and the other patient withdrew from the study prior to completion.

**Table 1:** Study population.

Gender	N	Age	N	Treatment	N
Female	6	30–49 yrs	3	SCT, allogeneic	3
Male	4	50–64 yrs	2	SCT, autologous	4
		65–80 yrs	5	CAR T-cell therapy	3

### Wearable RPM feasibility

The *a priori* established threshold for feasible use of wearable RPM devices for in-home monitoring was wear as intended by 6 patients over the course of the study, or 60%. Nine of 10 patients (90%) wore RPM devices as intended over the course of the study. The tenth patient withdrew from the study on the first day post-discharge, citing difficulty in device management between locations in their outpatient environment. Device wear behavior was confirmed through monitoring for on/off body alerts and time in off-body state. Total device wear time was calculated beginning after hospital discharge, with duration dependent on treatment type and post-treatment length of stay prior to discharge. For the 9 patients who completed the study, total wear time ranged from a low of 9.1 days to a high of 65.5 days. All 9 patients had high device wear compliance and wear time percentages of 95% or greater as calculated by minutes off-body versus minutes on-body during the monitoring period.

**Figure 1:**
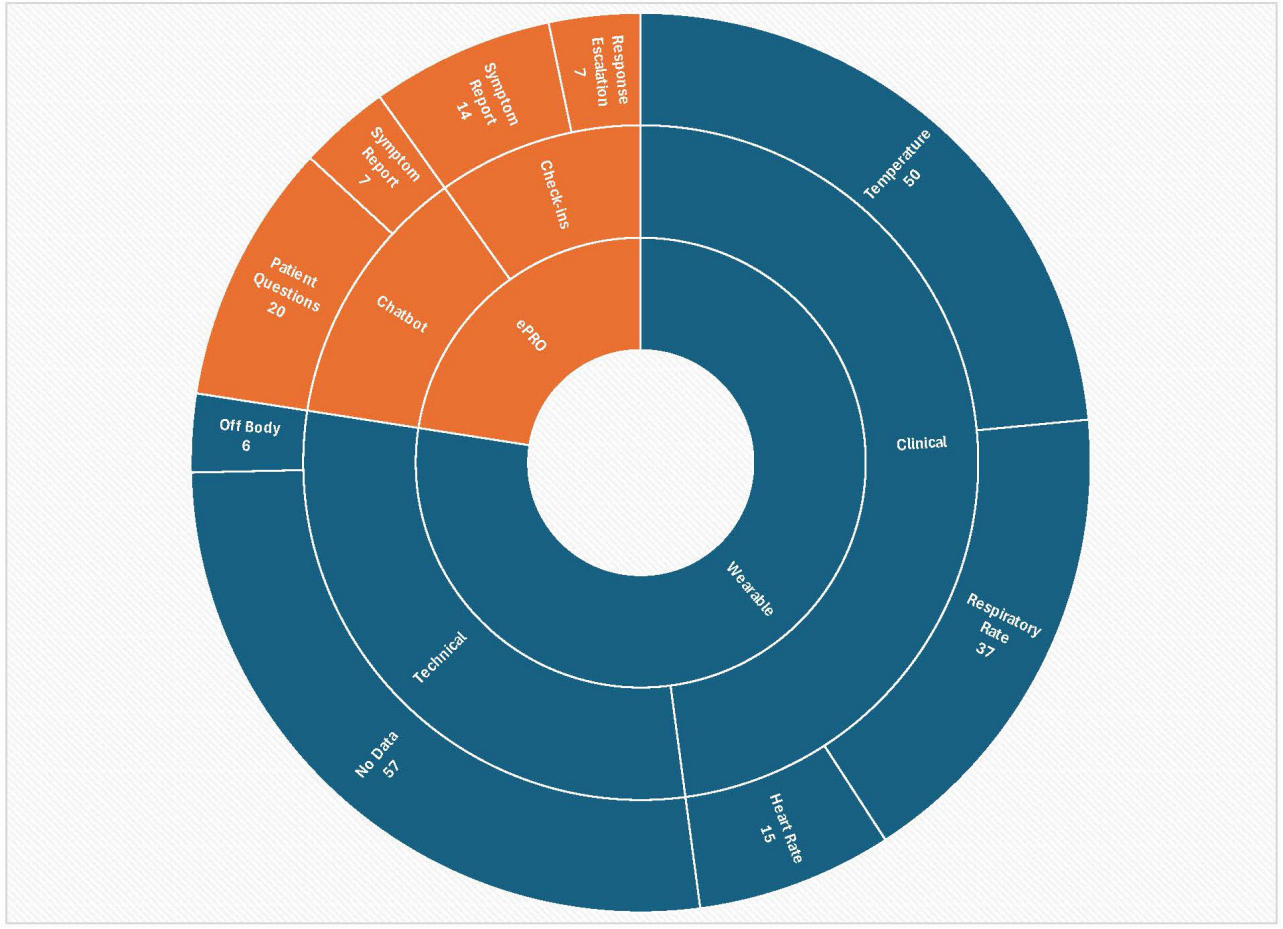
Clinical alerts received from wearable RPM and ePRO systems by source, type and volume.

A total of 171 alerts were received from wearables during the study period from patients receiving outpatient RPM. **Figure 1** depicts the distribution of alerts grouped in each ring by type from the center outward: first from the device, then whether clinical or technical, then by type of technical alert or type of clinical alert. Repeated alerts for the same event were possible if not resolved, thus the total number of alerts does not equate to the total number of events. Due to the small number of patients in each clinical subgroup, we did not analyze alerts by subgroup in order to avoid the possibility for a sicker patient with higher alert numbers to unintentionally bias results. Alerts that occurred after a patient had come off the study or while they were admitted to the hospital were excluded, resulting in 165 alerts for analysis. Among alerts received, over 60% represented clinical indicators (61.8%; n=102). The remaining 63 alerts were classified as technical, including off-body alerts (n=6) and issues with data transmission and synchronization (n=57; examples included patients being away from their home hub without their phones and thus out of range, along with alerts from two devices which experienced technical failure). All alerts were reviewed, including technical alerts. No delays in review from time of alert receipt by the VCC were noted. On instances where there were delays in data transmission due to patients being out of range, data was stored on the wearable and transmitted when patients returned to range. A total of 57 alerts of all types necessitated follow-up with patients, 15 alerts were referred to the patient’s clinical team for action, and 5 alerts required care in a clinical setting. One patient had 14 ER visits resulting in 6 hospitalizations; there were no other ER visits or hospitalizations needed among participants during the study period.

### Chatbot/ePRO symptom management feasibility

The *a priori* established threshold for success at engaging with the chatbot/ePRO system was engagement by 6 patients over the course of the study, or 60%. Nine of 10 patients (90%) engaged with the ePRO and chatbot system during the study. Patients also used all three aspects of the ePRO system over the course of the study. All 9 patients initiated contact with the chatbot by sending it at least 1 unprompted text message. Seven of 10 patients (70%) completed at least 1 ePRO request for a weekly health check survey, and 6 of 10 patients (60%) responded to chatbot-initiated check-ins (“review of systems,” or ROS) more than half the time. The number of ROS requests ranged from a low of 7 requests over an 11-day duration to a high of 51 requests over a 56-day duration. Although patients were enrolled in the study shortly prior to hospital discharge to allow for orientation, education, and baseline data collection from wearables, ROS requests were intended for outpatients only and did not begin until after hospital discharge. Further assessment with a larger population is needed to support inferential analysis to discern if patient engagement varies meaningfully over time, by interaction volume, or by clinical subgroup.

A total of 48 alert messages of various types that required responses from or action by VCC team members were received through ePRO, whether in response to check-in prompts or initiated by patients through the chatbot. Twenty-one represented symptom reports, of which 14 were received through check-in prompts and 7 through interactions with the chatbot. These results are shown in **Figure 1** categorized by type and source in each ring from the center outward: by ePRO, then whether in response to structured check-in prompts or from engagement with the chatbot, then by message type. “Response escalations” as a category represents responses to check-in prompts that were not symptom reports but still needed outreach from the team. Of all alert messages received through ePRO, 11 were referred to the patient’s clinical care team and 5 required follow-up care in a clinical setting. Common symptoms reported were fever or chills, nausea, dizziness, rash, cough, swelling, and pain. Nearly half of chatbot/ePRO alerts (n=20) represented questions asked by patients. No technical issues were reported with chatbot function, whether with inbound messaging, outbound prompts to patients, or alerts.

### DHT acceptability (user experience)

All surveyed (n=6) and interviewed (n=8) patients perceived that the wearable RPM device and the ePRO chatbot system were easy to use. Half (n=4) of interviewed patients specifically noted that the monitoring provided them with a sense of connectedness and a positive feeling that someone was looking out for them, 5 perceived the care program to be a good idea in general across interviews and surveys, and all 6 surveyed patients reported that RPM made them feel more cared for by their health care team. Example illustrative quotes from patients are included in **Table 2**.

**Table 2:** Patient experience quotes.

Topic	Quotes
Ease of use	“Like I said, they were easy to use. I mean, you really didn’t have to do anything once it got set up.” “It just was plug and play and stick that thing on and just respond to a text once in a while. And I don’t know what could make it easier.” “It’s very easy, it’s very lightweight. You know, when we did have to replace it or take it off for charging, it was very easy to do that. It’s easy to sync up with my phone, it was easy to sync up with the sync device, the button that we had in … here. So it’s very user friendly.”
Overall perception	“It seemed like a really good program.” “And I think it’s good for people if they want to do it. I mean, you get home and you feel like stuff can just go bad. And we felt like we were connected to the hospital. So again, I think that’s the biggest plus and I got some good advice and I got some good help.”
Device	“So … it’s pretty unobtrusive little device and was easy to plug and play. And I think, I think it’s a, it’s a good liaison, too. If you have issues … That’s comforting to me.” “We went somewhere, you know, for two or three hours or something? …and I would get a message on my phone, that it hadn’t been receiving any data for [the] whole period of time.” “I liked the fact that for the most part, it was picking up the information. And if it picked up information, like a high heart rate, I usually would get a call to say, ‘Hey, your heart rate seems or are you doing okay.’ And so it seemed like it was giving some pretty good biofeedback.”
Chatbot	“It certainly helped … after hours, it was so nice. Because calling some you know, they’ll say, oh, just call. Well, sometimes it’s not so easy after hours to get somebody … instead with [the chatbot], we just hook them up.” “[The chatbot] needs a little help in his programming to clarify things. Nuances … I mean, he was very basic with his questions.”
Suggestions	“Better understanding [of] what you’re trying to ask? Or maybe a system if [the chatbot] doesn’t understand that, then there’s a process to get a hold of a human who can.” “I don’t know if it improves it. But it called me a couple of times pretty early in the morning about elevated body temperature and take your temperature. And I thought that was a little on the prudent side, because I really didn’t have a fever. So that, you know, just kind of an inconvenience, but I know it’s just trying to do the right thing.”

For the most part, patients found the wearable RPM device unobtrusive (n=4), but some noted issues with sticker adhesives (n=3), skin irritation (n=2), synchronization issues (n=2), general discomfort while wearing (n=1) and in two cases the need to replace the wearable early due to technical failure. A single wearable was intended for use per patient for the duration of the study; technical failure required replacement. Patients did not otherwise make particular reference to needs for technical support. Patients also noted concerns about overnight alerts (n=4); two noted that text messages did not wake them and they would not have been able to address needs in a timely fashion, and two noted that the overnight temperature elevations that they received calls about were not accurate to thresholds of concern due to them being under covers and skin temperature being falsely elevated.

Patients were able to tell the difference (n=4) between AI chatbot interactions and human interactions in the chatbot system, especially when the chatbot didn’t seem to understand or wasn’t able to accept answers to questions after a certain period of time (n=2). Patients found the chatbot messaging to be helpful (n=4), reporting that they liked being able to ask questions, the all-hours nature of being able to receive either an automated or human response through the chatbot, and that they also liked the daily check-ins – although some (n=2) felt that they received too many messages.

Suggestions offered from patients included making the wearable RPM’s mobile app easier to read and having it contain more information and include historical data, addressing phone battery depletion attributable to app use, allowing the lights on the RPM hub to be dimmable or turned off, and making the wearable device button easier to press (n=1 each). Patients also shared questions and suggestions to improve education and program awareness (n=5), including requests to engage caregivers more comprehensively in program planning and care (n=3) and for more detailed information about which services were part of which program (n=3).

Among caregivers, 5 of 6 survey respondents found RPM useful, felt it helped with the stress of caregiving, and reported feeling their loved one was well cared for. Suggestions for improvement from caregivers included requests for more information, opportunity to reduce device wear if the patient was doing well, and longer battery life.

Among providers, 6 of 8 survey respondents felt RPM improved quality of care and detection of fever, infection, and symptoms among their patients. The same number of providers (n=6) were satisfied with the communication from the virtual care team and felt that RPM should be used more often to manage care at home. Provider suggestions included improving the communication process between the virtual care team and the patient’s usual care team and refining alert thresholds to avoid false or overly sensitive alerts. Both issues were perceived as increasing provider burden.

## Discussion

This study sought to determine the feasibility and acceptance of a technology-supported, in-home program to provide patient-centered care for patients with cancer. Understanding these measures as composites and by their inherent aspects, including if there were different types of interactions that patients found more value in or were either more or less willing to interact with (e.g., if patients liked having the ability to ask questions through the chatbot but preferred not to answer ROS check-ins), was essential to achieve more comprehensive insight regarding the overall program. The program was found to be acceptable by 9 patients who had received SCT or CAR T-cell therapy, their caregivers, and their providers, all of whom reported positive experiences. Thresholds established ahead of time to define both technical and operational feasibility for RPM and ePRO based on the number of patients who wore RPM devices as intended and the number of patients who engaged with all aspects of the ePRO system (chatbot, check-ins, ROS) over the course of the study were met and surpassed, indicating the feasibility of using DHTs for patient monitoring and symptom reporting and management. These findings are consistent with other similar studies examining both the feasibility and effectiveness (36–39) of RPM and ePRO at improving care for cancer patients, which further supports the promise of such technology support systems for improving care management and health outcomes.

One benefit of conducting feasibility and functionality testing at the pilot level is the ability to identify and address issues and challenges that would otherwise affect success and sustainability at scale. Health care is a complex adaptive system, in which pressures in one area of a workflow can cause unexpected and unintended consequences at another. Technical and operational feasibility challenges could cause failure of either or both larger clinical trials or implementation of a RPM program in clinical practice if not identified and accounted for in advance, and this study is an important part of that preparation. Similarly, user experiences are critically important to successful solution adoption, and understanding barriers and facilitators for users at all levels is an essential part of ensuring good user acceptance. Findings from this study will support future improvements in care processes and clinical integration. Successful integration of outpatient services with those delivered in clinical practice settings requires tailoring and adaptation of existing workflows, good coordination across care teams, and both functional and experiential interoperability across electronic medical record systems.

The implications of DHTs being found feasible and acceptable for use to support better-managed care at home are extensive and multifaceted. Reducing the need for utilization of acute care in settings such as the emergency room and hospital may not only lower burden on patients and caregivers but also on providers and the health system. Moreover, increased feelings of reassurance and connection to care resulting from the knowledge of the RPM itself even in the absence of averted infection or CRS events may increase satisfaction and reduce psychosocial stress among patients and caregivers, which can improve health outcomes (40). Using AI solutions to augment human-provided care offers the potential to support routine patient needs in an empathetic manner while appropriately engaging clinicians for more complex questions or for clinical evaluation (41). At the same time, opportunities remain to refine alerting criteria and thresholds to reduce burden and avoid alert fatigue while still ensuring patient safety. Examples include the potential need to tailor and refine clinical alerts to be sensitive to factors such as artificially elevated temperatures due to blankets at night and temporarily elevated heart rates or respiratory rates due to activities that result in more physical exertion from these patients versus the general population, such as walking from a parked car to a clinic appointment. Similarly, “no data” alerts attributable to patient behaviors such as not keeping their phones with them or not having the data synchronization app activated on their phones at all times mean that alternatives must be explored to enable easier transmission without unintentionally limiting patients’ activities. Possibilities include options such as placing data transmission hubs in clinic environments to close the gap during longer-duration clinic appointments or finding other ways for the devices to transmit (eg, smarter wearables). These factors are critical to understand and account for in achieving acceptable and reliable alerting and response, particularly given the time sensitivity for intervention in this patient population.

The study team also explored the potential for providing home health care such as laboratory draws, physical assessments, and intravenous fluids and antibiotics to study patients. However, this initial exploration did not meet the threshold to assess for feasibility due to challenges with obtaining timely cyclosporine and tacrolimus levels, cytomegalovirus PCR quantification, and limitations due to thrombocytopenia and administration of in-home transfusions for this patient population through routine home health services, as opposed to a “hospital at home” acute care approach. Further challenges were identified with regard to intersystem coordination, communication and integration for these types of services. Future assessment and evaluation of such options remains important to improve at-home care to support patients with cancer by further expanding the services safely available in the home setting.

As a feasibility study, this work was not intended to support inferential analysis or empirically validated conclusions about direct impact on health outcomes. Results should be interpreted for the study population rather than as broadly representative, with inherent limitations due to study size and the single health system context. Next steps planned include bringing the solution to scale in the current practice setting and assessing the impact with larger numbers of participants on infection rates and infections avoided, other cancer-directed therapy complications, symptom management, resources required to implement, health economics associated with technology costs and personnel for monitoring, and safety of at-home interventions. Additional future research with multiple sites and practice settings is also desirable to inform broader practice. This potential improvement in infection and neurotoxicity outcomes in association with avoidable hospitalizations, improvements in patients’ morbidity, mortality and quality of life, in addition to reductions in healthcare cost and staff and facility burden, holds the potential to be transformative for clinical practice in oncology and beyond.

## Data Availability

The raw data supporting the conclusions of this article will be made available by the authors, without undue reservation.
